# Surface and Bulk Defect Passivation in MAPbI_3_ Perovskites with Daminozide: Effects on Carrier Dynamics and Mobility

**DOI:** 10.1002/advs.202500530

**Published:** 2025-05-08

**Authors:** Junhan Xie, Di Li, Haozheng Li, Bo Peng, Qinye Bao, Jiaming Jiang, Bo Li, Weimin Liu

**Affiliations:** ^1^ School of Physical Science and Technology ShanghaiTech University Shanghai 201210 P. R. China; ^2^ Department of Physics East China Normal University Shanghai 200062 P. R. China

**Keywords:** MAPbI_3_ perovskite, mid‐IR emissive trapping state, surface and bulk defect passivation, transient absorption microscopy, transient mid‐IR spectroscopy

## Abstract

Metal halide perovskite semiconductors are highly valued for their outstanding optoelectronic properties. However, the high density of intrinsic defect states in their polycrystalline thin films on the surface and within the bulk poses a significant challenge by diminishing carrier mobility and lifetime, thus hindering device performance. This study reveals a previously unidentified mid‐IR emissive trapping state in MAPbI_3_ that differs from conventional Shockley‐Read‐Hall (SRH) defects, exhibiting unique surface‐localized characteristics detectable through transient mid‐IR spectroscopy. A dual‐function passivation strategy using daminozide (DA) is developed, where the interlayer selectively passivates mid‐IR‐active surface defects while the additive mitigates bulk SRH defects through carbonyl‐Pb^2^⁺ coordination. This passivation strategy yields remarkable improvements in carrier dynamics, increasing diffusion constants from 0.135 to 0.165 cm^2^ s⁻¹ and significantly enhancing the device performance, including open‐circuit voltage and power conversion efficiency. These findings highlight the crucial importance of addressing both surface and bulk defects to optimize the optoelectronic properties of perovskites.

## Graphical abstract

This study identifies a novel surface defect in MAPbI_3_ perovskite that emits mid‐infrared light, distinct from Shockley‐Read‐Hall (SRH) defects. By applying daminozide as a surface passivation layer and bulk additive, both defect types are effectively suppressed. This dual‐function passivation strategy enhances electron mobility and improves device performance, including open‐circuit voltage and power conversion efficiency.

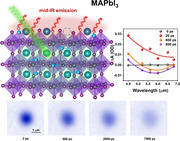

## 1 Introduction

Metal halide perovskite semiconductors have garnered widespread attention globally due to their remarkable optoelectronic power conversion efficiency (PCE), extended carrier lifetime, and high carrier mobility.^[^ ^1^ ^]^ Unlike perovskite single crystals, solution‐processed perovskite polycrystalline thin films exhibit a high density of intrinsic defect states at the interfaces and grain boundaries during film growth.^[^ ^2^ ^]^ These defects include uncoordinated Pb^2+^ ions, I^−^ ions, and their antisite dangling bonds.^[^ ^3^ ^]^ The presence of these defects significantly impairs carrier transport by increasing recombination losses and reducing the effective carrier lifetime. As a result, defects contribute to reduced device performance and accelerated degradation, limiting the potential of perovskite‐based devices.^[^ ^4^ ^]^

Surface and bulk defects in perovskites are typically associated with trap‐assisted recombination, where charge carriers (electrons and holes) are captured by defect states, reducing the overall efficiency of the devices.^[^ ^2^, ^5^ ^]^ Defect passivation to reduce unproductive charge recombination is an effective strategy to increase the PCE of polycrystalline perovskite photovoltaics.^[^ ^6^ ^]^ Previous studies have highlighted various approaches to defect passivation, including the use of small molecules and interlayers to reduce defect density and suppress trap‐assisted recombination pathways.^[^ ^4^, ^7^ ^]^ A promising candidate for this purpose is daminozide (DA), a small organic molecule that can act both as an interlayer and additive passivator.^[^ ^1^, ^8^ ^]^ A remarkable power conversion efficiency of 22.15% was achieved for polycrystalline MAPbI_3_‐based p‐i‐n structural perovskite solar cells with DA passivation.^[^ ^1b^ ^]^ However, the precise mechanism by which DA modifies the defect landscape and its effects on device performance remain insufficiently understood. To address these challenges, it is essential to understand the impact of defect states on carrier dynamics.

This study examines how DA passivates defect states in polycrystalline MAPbI_3_ perovskite films. We investigate the effects of incorporating DA both as an interlayer and as an additive on the temporal and spatial dynamics of carrier diffusion in perovskite devices. Using transient absorption (TA) spectroscopy, time‐resolved photoluminescence (TRPL), and transient mid‐infrared (mid‐IR) spectroscopy, we assess the impact of DA passivation on defect states. Although conventional techniques like TRPL, thermal admittance spectroscopy, and sub‐bandgap external quantum efficiency measurements have effectively quantified the reduction of defect states by the passivation, they mainly identify defects linked to non‐radiative transitions and cannot isolate mid‐infrared emissive states.^[^ ^9^ ^]^ In our experiments, transient mid‐IR spectroscopy directly probes mid‐IR emissions, leading to the discovery of a novel long‐lived mid‐IR emissive trapping state at the MAPbI_3_ perovskite film surface—distinct from the deep trap state associated with Shockley‐Read‐Hall (SRH) recombination. Overall, DA‐induced passivation of both surface and bulk defects significantly enhances the perovskite's photoelectrical properties by reducing trap‐assisted recombination and slowing carrier recombination dynamics. Furthermore, transient absorption microscopy (TAM) reveals improved carrier mobility and morphological modifications induced by DA, with results strongly correlated to device efficiency.

## 2 Results and Discussion

**Figure 1:**
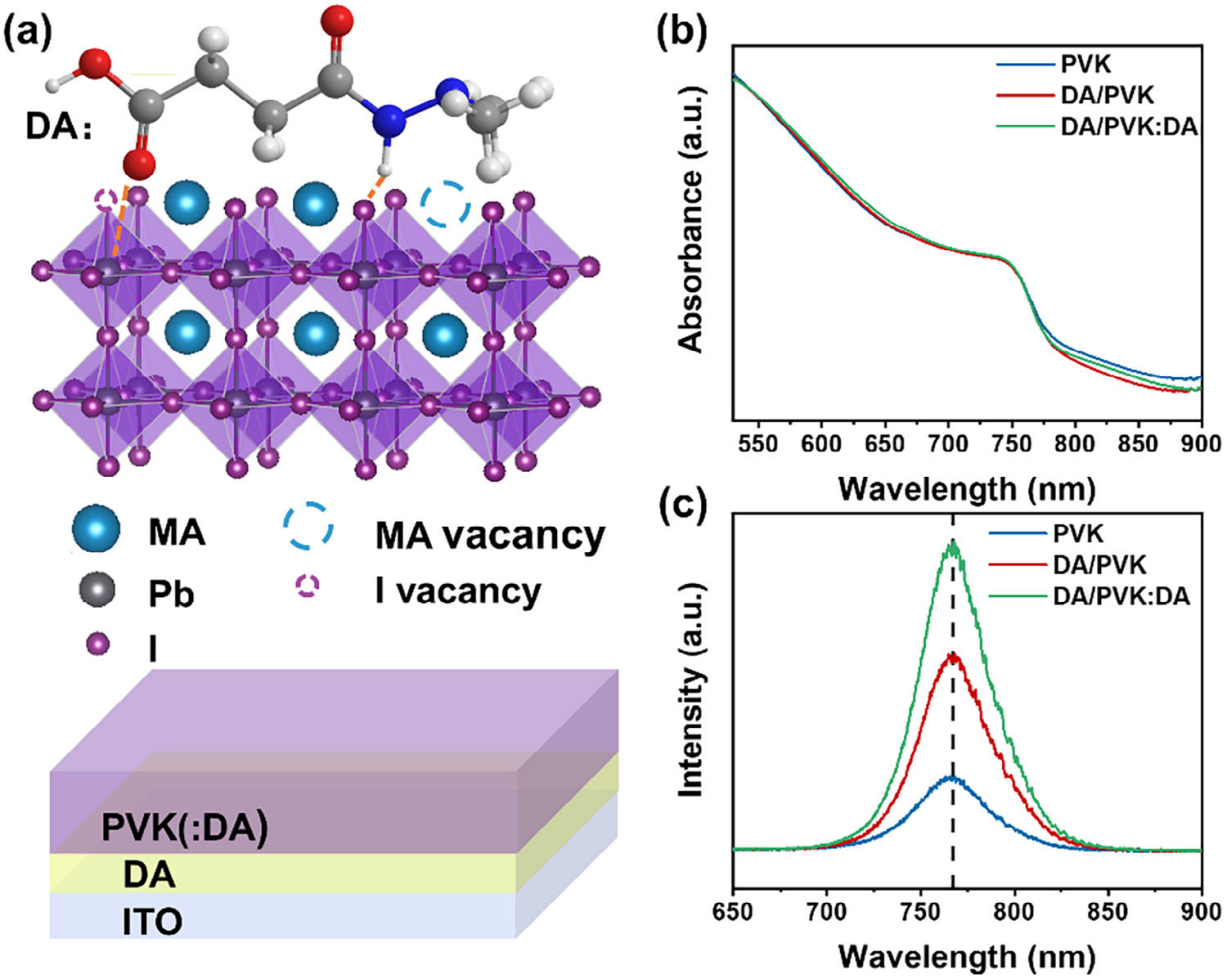
a) Top: Schematics of the interactions between DA and MAPbI_3_, Bottom: schematics of perovskite film architecture; b) UV–vis absorption spectra and c) steady‐state photoluminescence of perovskite thin films.

To investigate the role of defect states in MAPbI_3_ perovskite, we selected traditional polycrystalline films (PVK) as the baseline. In this study, DA was employed both as an interlayer to passivate surface defects and as an additive to mitigate bulk defects. This approach yielded three distinct perovskite samples: pristine PVK, DA as an interlayer (DA/PVK), and DA as both an interlayer and additive (DA/PVK: DA). The device architecture of DA/PVK, illustrated in **Figure** **1**a, follows an ITO/DA/perovskite configuration. The UV–vis absorption and photoluminescence (PL) spectra of the perovskite samples are presented in Figure 1b,c. The UV–vis absorption spectra reveal the bandgap of all samples at ≈1.58 eV (785 nm). Upon the application of DA, the PL yield is significantly enhanced, combined with the decrease in the defect‐related sub‐band gap absorption tail (800–900 nm),^[^ ^10^ ^]^ which indicates the effective passivation of defect states. Passivation did not alter the conduction band (CB) and valence band (VB) structures, as reflected by the consistent bandgap and PL peak across the samples.

### 2.1 Identification of Mid‐IR Emissive Trapping State in Pristine PVK

#### 2.1.1 TA and TRPL Showcase a Nonradiative Sub‐Bandgap Trapping State that Differs from the SRH‐Related Deep Defects

**Figure 2:**
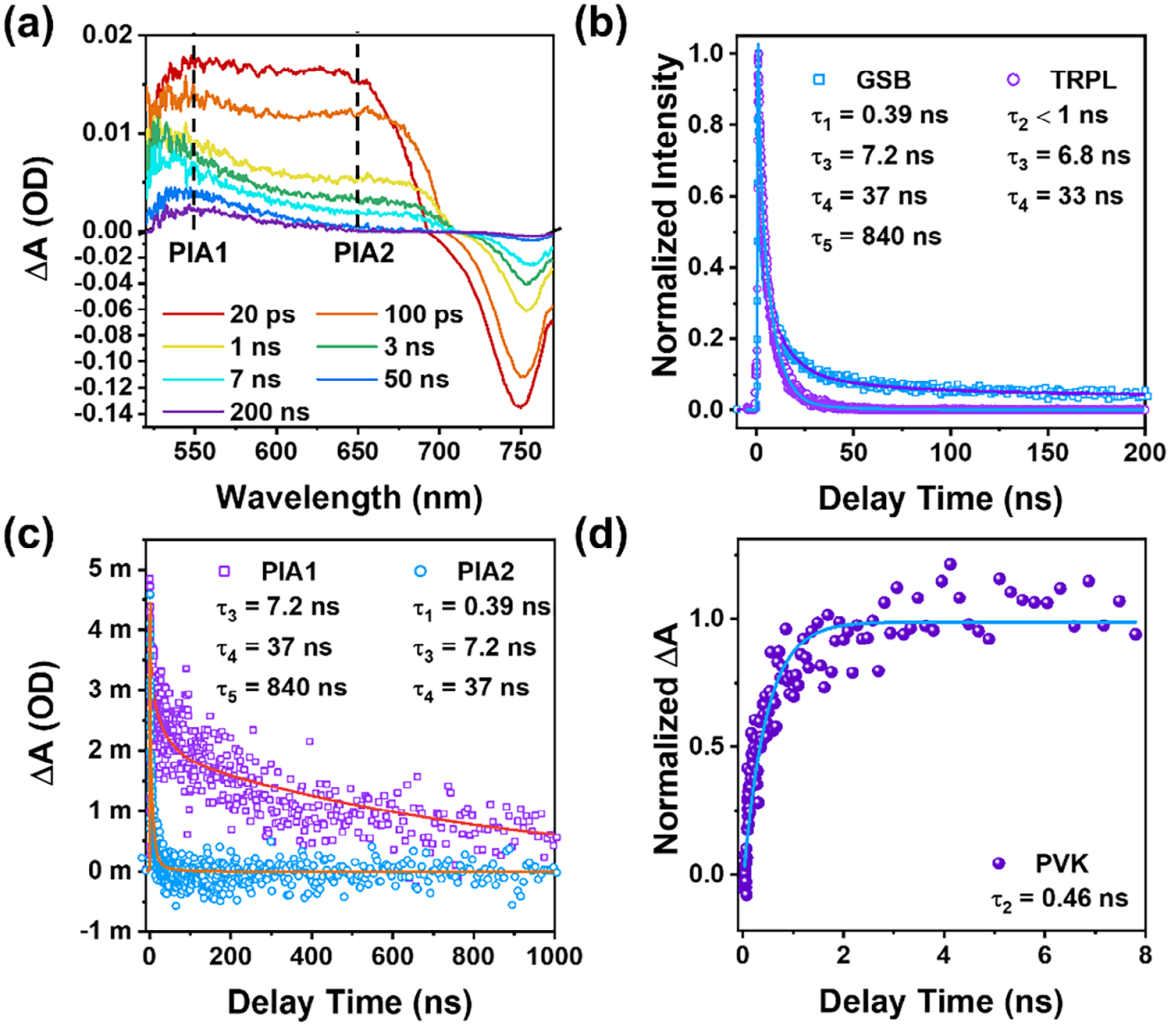
a) Transient absorption spectra of PVK at the selected delay time, with PIA1 and PIA2 band depicted by dash lines; b) Normalized kinetic trace of GSB signal from TA and TRPL (probe at 770 nm) from TCSPC in PVK; c) Kinetic trace of PIA1 and PIA2 bands in PVK; d) The normalized rising dynamics of sub‐bandgap trapping state within PIA1 band of PVK.

TA spectroscopy was employed to probe the defect state dynamics. The TA spectrum of PVK under 515 nm excitation with an excitation power density of 128 µJ cm^−^^2^, shown in **Figure** **2**a and Figure S2 (Supporting Information), reveals complex excited‐state dynamics across the spectral range of 500–800 nm, with temporal evolution extending up to the microsecond (µs) timescale. These dynamics include a negative ground‐state bleach (GSB) signal centered at ≈755 nm and a broad positive photoinduced absorption (PIA) signal spanning the spectral range 500–700 nm.^[^ ^11^ ^]^ As shown in Figure 2b, the GSB dynamics are fitted using a multi‐exponential decay model, yielding four characteristic lifetimes: τ_1_  =  0.39 ns (69%), τ_3_ = 7.2 ns (24%), τ_4_ = 37 ns (5%), and τ_5_ = 840 ns (2%). The shortest lifetime, τ_1_ (0.39 ns), is attributed to inter‐band Auger recombination, associated with the slow‐down cooling of hot carriers.^[^ ^12^ ^]^ The longer lifetimes, τ_3_ (7.2 ns) and τ_4_ (37 ns), correspond to electron‐hole bimolecular recombination and deep trap‐assisted SRH recombination, respectively, as observed in TRPL measurements in Figure 2b.^[^ ^13^ ^]^ The longest lifetime, τ_5_ (840 ns), though contributing only 2% to the total TA signal, is notably longer than typical SRH recombination lifetimes. Remarkably, this long‐lived τ_5_ component is absent in the TRPL dynamics probed at 770 nm, as shown in Figure 2b.

Interestingly, as shown in Figure 2a, the TA spectrum of PVK reveals two distinct PIA bands centered at 550 nm (PIA1) and 650 nm (PIA2). PIA2 band exhibits three exponential decay lifetimes: τ_1_ = 0.39 ns, τ_3_ = 7.2 ns, and τ_4_ = 37 ns, see Figure 2c, indicating Auger, bimolecular, and SRH recombination from the CB. In contrast, PIA1 demonstrates a significantly longer decay lifetime, shown in Figure 2c, matching the τ_5_ (840 ns) observed in GSB dynamics. These findings indicate that the PIA1 band likely arises from a sub‐bandgap trapping state located between the VB and CB, rather than directly from the CB.

The dynamics of the rising edge associated with this trapping state within the PIA1 band offer significant insights to elucidate the sub‐bandgap trapping state further. As shown in Figure 2a, the spectral overlap between the PIA1 and PIA2 bands obscures the initial rising dynamics of the PIA1 band. This overlap was resolved by subtracting the PIA2 dynamics at ≈650 nm from the PIA1 dynamics at ≈550 nm. As shown in Figure 2d, the isolated rising dynamic of the PIA1 band is fitted with a single exponential rise dynamic of τ_2_ = 0.46 ns. Note that the τ_2_ is close to the lifetime of the Auger process (τ_1_ = 0.39 ns), making it challenging to differentiate from them in TA spectra. Notably, the TRPL dynamics of PVK require three lifetimes (<1, 6.8, and 33 ns) for the best fit (see Figure 2b). It is straightforward to attribute τ_3_ = 6.8 ns and τ_4_ = 33 ns to the electron‐hole bimolecular recombination and SRH recombination, which is consistent with the TA results. However, the first decay lifetime of <1 ns is not well identified due to the instrument response function (≈500 ps) in the TCSPC measurement. Given the low excitation power density (4 µJ cm^−2^) used in the TCSPC experiment, this first short lifetime is more likely associated with the decay dynamic (τ_2_) from CB to form the sub‐bandgap trapping state rather than the Auger process (τ_1_), as the latter is negligible under such low carrier density conditions.^[^ ^14^ ^]^ In summary, the sub‐bandgap trapping state associated with the PIA1 band exhibits a rise time of 0.46 ns (τ_2_) and a decay time of 840 ns (τ_5_). This newly observed deep defect state may reside between the CB and VB and play a critical role in the trapping state recombination.

#### 2.1.2 Transient Mid‐IR Spectra Identify this Sub‐Bandgap Trapping State as a Mid‐IR Emissive Trapping State

The previous report has indicated that in MAPbBr_3_, a near‐IR defect emission center at 1000 nm has been observed, with a decay lifetime on the order of hundreds of nanoseconds.^[^ ^15^ ^]^ This decay lifetime closely matches the longest recovery lifetime obtained in the GSB signal in TA spectra.^[^ ^15b^ ^]^ Comparable experimental findings were noted in MAPbI_3_, however, the 1000 nm near‐IR emission is absent. Despite this, the GSB dynamics monitored through TA still reveal the presence of a trapping state in MAPbI_3_ that decays on µs timescales,^[^ ^15b^ ^]^ similar to our findings. This suggests that the trapping state in MAPbI_3_ and MAPbBr_3_ share similar physical pathways. Nonetheless, the question of whether the trapping state in MAPbI_3_ is insufficiently near‐infrared emissive or shifts to other infrared wavelength regions, resulting in non‐radiative recombination in the visible region, remains unresolved and warrants further investigation.

**Figure 3:**
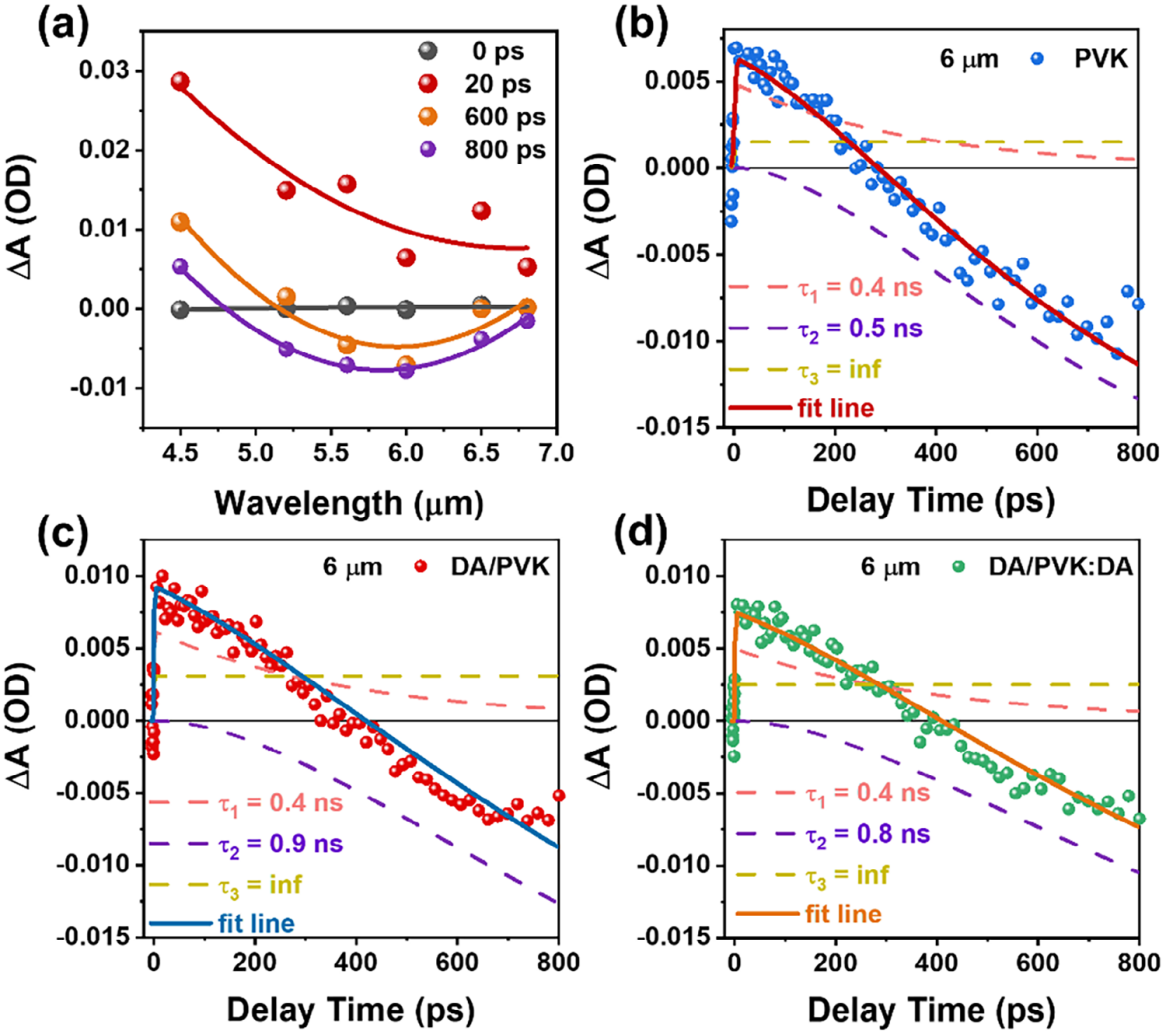
a) Transient mid‐IR spectra of PVK at t = 0, 20, 600, and 800 ps; Kinetic trace of PVK b), DA/PVK c), and DA/PVK: DA d) at 6 µm, the fitting curve (solid line) is comprised of three components (pink dash line: ESA from Auger recombination, yellow dash line: ESA from electron‐hole bimolecular recombination, and purple dash lines: STE form mid‐IR emissive trapping state).

To gain deeper insights into this long‐lived sub‐bandgap trapping state, we employed transient mid‐IR spectroscopy, using 400 nm excitation and probing across the wavelength range of 4 to 7 µm, as shown in **Figure** **3**a. The results reveal an initial broad excited‐state absorption (ESA) band across this entire wavelength range, corresponding to the population of electrons in the CB.^[^ ^16^ ^]^ After several hundred picoseconds delay, a negative absorption band emerges, centered at ≈6 µm. This negative signal originated from stimulated emission (STE) at 6 µm and is attributed to mid‐IR emission from iodide (I) derivatives forming a trapping state in MAPbI_3_, as previously reported studies.^[^ ^16a^ ^]^ This assignment is further supported by X‐ray photoelectron spectroscopy (XPS) and Fourier transform infrared spectroscopy (FTIR) analyses presented in Section 2.2.3 and Figure S7 (Supporting Information). Notably, this Mid‐IR emissive band is not observed when iodine was replaced by bromine (Br) in perovskite,^[^ ^16a^ ^]^ likely due to the Br‐based perovskite blueshift of the emission of this trapping state to 1000 nm, in line with the 1000 nm near‐IR emission observed in MAPbBr_3_.^[^ ^15a^ ^]^

The dynamics of the STE signal in PVK were analyzed by fitting the transient amplitude at 6 µm, plotted in Figure 3b. This was modeled using a negative STE component (purple dash line) combined with a positive ESA component (pink dash line and yellow dash line), which includes Auger recombination (τ_1_ = 0.4 ns) and a positive infinite lifetime component (bimolecular recombination τ_3_, set as infinite due to the 800 ps time window). The negative STE component was found to have a rise lifetime of ≈0.5 ns, consistent with the τ_2_ = 0.46 ns rise time observed in the TA experiment (see Figure 2d), confirming that the trapping dynamic from the CB to the sub‐bandgap trapping state (mid‐IR emissive trapping state) is ≈0.46 ns. However, the decay dynamics of this trapping state could not be fully resolved due to the limited time window of the transient mid‐IR measurements. The analysis indicates that the trapping state predominantly captures electrons, differentiating it from SRH recombination. It is characterized as non‐radiative within the visible wavelength spectrum, yet it exhibits emissive properties in the mid‐IR range.

To date, TA and transient mid‐IR spectra for pristine PVK show a distinctive mid‐IR emissive trapping state in pristine PVK, characterized by a rise time of 0.46 ns and an extended decay lifetime of 840 ns. This state is absent in the TCSPC spectra within the visible range but is evident in the mid‐IR range, suggesting the formation of a trapped state by I derivatives in PVK.^[^ ^16a^ ^]^ The precise nature of this trapping state, whether linked to bulk or surface defects, remains undetermined. A viable strategy to elucidate this issue involves altering the density of the state (DOS) of the defects through passivation. DA serves as an effective agent for defect passivation, applicable both as an interlayer and as an additive, targeting defects in both surface and bulk regions^[^ ^1b^ ^]^ (see Supporting Information Section S1).

### 2.2 Identification of Surface and Bulk Defects via DA Interlayer and Additive Passivation

To investigate the mid‐IR emissive trapping state, we examined two passivated perovskite samples: DA/PVK (interlayer passivation) and DA/PVK:DA (interlayer and additive passivation). The TA spectra (Figure S3, Supporting Information) at a 4 ns delay reveal a striking reduction in the PIA1/PIA2 peak ratio from 4:1 in pristine PVK to 1:1 in both passivated systems (Figure S4, Supporting Information), indicating effective suppression of mid‐IR emissive trapping states and a corresponding decrease in their DOS. This spectral evolution provides clear evidence for defect passivation efficacy.

#### 2.2.1 Surface Defect Passivation

The interlayer passivation effect was quantitatively analyzed through time‐resolved measurements. DA/PVK exhibits a characteristic trapping time constant (τ_2_) of 1.0 ns (Figure S5a, Supporting Information), which is double that of the pristine PVK (0.46 ns). This prolonged trapping time directly evidences the reduced surface defect DOS, as the capture lifetime of carriers by defect states is inversely proportional to the available trap states. Complementary evidence comes from the τ_2_  =  0.9 ns rise time observed in transient mid‐IR spectra in DA/PVK at ≈6 µm (Figure 3c), matching the TA‐derived τ_2_ (Figure S5a, Supporting Information). Notably, the similar τ_2_ values for DA/PVK (Figure 3c; Figure S5a, Supporting Information) and DA/PVK:DA (Figure 3d; Figure S5b, Supporting Information) indicate that additive incorporation minimally affects surface trapping states, confirming the surface‐localized nature of these mid‐IR emissive defects. This conclusion is further supported by the identical PIA1/PIA2 ratios in both passivated systems (Figure S4, Supporting Information).

Furthermore, the surface passivation effect extends to SRH recombination processes, as evidenced by TRPL measurements showing the SRH lifetime (τ_4_) increasing from 33 ns (PVK) to 52 ns (DA/PVK) (Figure S6a, Supporting Information), while GSB signal analysis in TA spectra (Figure S6b, Supporting Information) provides additional verification. This enhancement demonstrates that DA interlayer treatment effectively mitigates both mid‐IR emissive traps and conventional SRH‐active surface defects.

#### 2.2.2 Bulk Defect Passivation

The DA additive strategy reveals distinct bulk passivation effects. Notably, the PL intensity from DA/PVK:DA increases significantly compared to DA/PVK (see Figure 1c), indicating that the additive reduces DOS of the trapping state in the bulk material. This observation is supported by the extended SRH recombination lifetime (τ_4_) in DA/PVK:DA measured through both TRPL and TA spectroscopy (Figure S6, Supporting Information), suggesting that bulk passivation impacts SRH‐related deep defects. The bulk passivation through DA additive is further corroborated by grain size enlargement observed in scanning electron microscopy (SEM) (Figure 6f), which reduces grain boundary defect density. These results collectively demonstrate that while surface passivation dominates mid‐IR trap suppression, bulk passivation plays a crucial role in mitigating SRH‐active defects.

#### 2.2.3 Molecular‐Level Passivation Mechanism and Carrier Recombination Model

Our experimental results demonstrate DA is an effective passivator for both mid‐IR emissive trapping states and SRH defects in PVK. To elucidate the underlying molecular mechanism, we performed XPS and FTIR analyses to investigate the coordination between DA's carbonyl groups and uncoordinated Pb^2^⁺ ions. The XPS results, plotted in Figure S7a (Supporting Information), reveal that the 4f core level of Pb in PVK: DA (DA additive) exhibits a 0.2 eV shift toward higher binding energy compared to pristine PVK, confirming the interaction between uncoordinated Pb^2^⁺ ions and the DA molecule. This interaction is further substantiated by FTIR spectroscopy (Figure S7c, Supporting Information), where the C═O stretching vibration undergoes a 20 cm⁻¹ blueshift (1625 cm^−1^ in DA→1645 cm⁻^1^ in PVK:DA), characteristic of coordination bond formation between the carbonyl lone pair electrons and Pb^2^⁺ ions.^[^ ^17^ ^]^ These molecular‐level insights suggest that the defect states of PVK originate from uncoordinated Pb^2^⁺.^[^ ^18^ ^]^ The analogous passivation effect observed at the perovskite interface in DA/PVK samples suggests that the interlayer modification follows a similar coordination mechanism. Therefore, the mid‐IR emissive defects, which can be passivated by DA interlayer, originate from uncoordinated Pb^2+^ resulting from I deficiencies at the surface, such as V_I_ or Pb_I_ antisites.^[^ ^18^ ^]^

This discovery prompts further investigation into whether analogous mid‐IR emissive states exist in other iodide‐based perovskites. Supporting this hypothesis, Figure S8 (Supporting Information) exhibits the transient mid‐IR trace of FAPbI_3_ at the selected probe wavelength, where a pronounced STE signal at 5.6 µm confirms the existence of a trap state. This finding provides further evidence that the mid‐infrared emission state is intrinsically linked to I‐related defects in halide perovskites.

**Figure 4:**
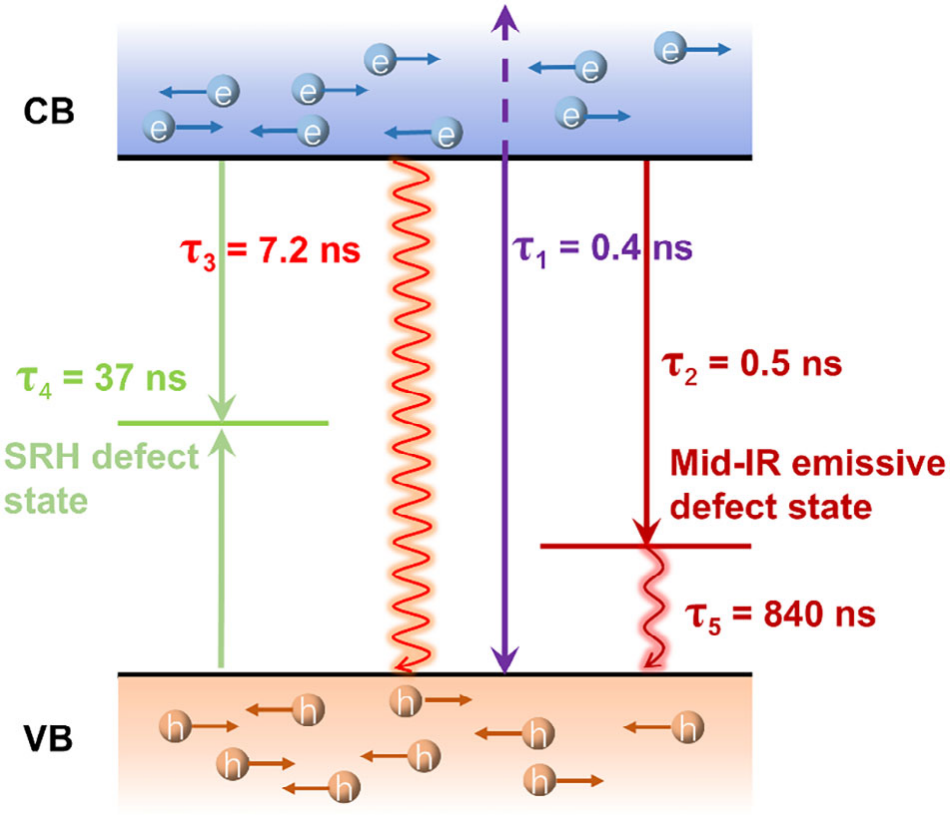
Schematic representation of the carrier recombination mechanism in MAPbI_3_ perovskite films.

Based on our analysis, we developed a carrier recombination model, identifying five distinct recombination lifetimes, as shown in **Figure** **4**. The fastest, τ_1 _= 0.4 ns, is linked to Auger recombination at high carrier densities. The intrinsic fluorescence lifetime of perovskite, τ_3 _= 7.2 ns, remains unaffected by passivation. The SRH recombination lifetime, τ_4_ = 37 ns, is influenced by both DA additive and interlayer passivation. Mid‐IR emissive deep defect states capture carriers from CB in τ_2_ = 0.5 ns, primarily affected by the DA interlayer, and eventually return to the valence band with a time constant of τ_5_ = 840 ns.

### 2.3 Impact of Defect Passivation on Carrier Spatiotemporal Diffusion and Device Performance

**Figure 5:**
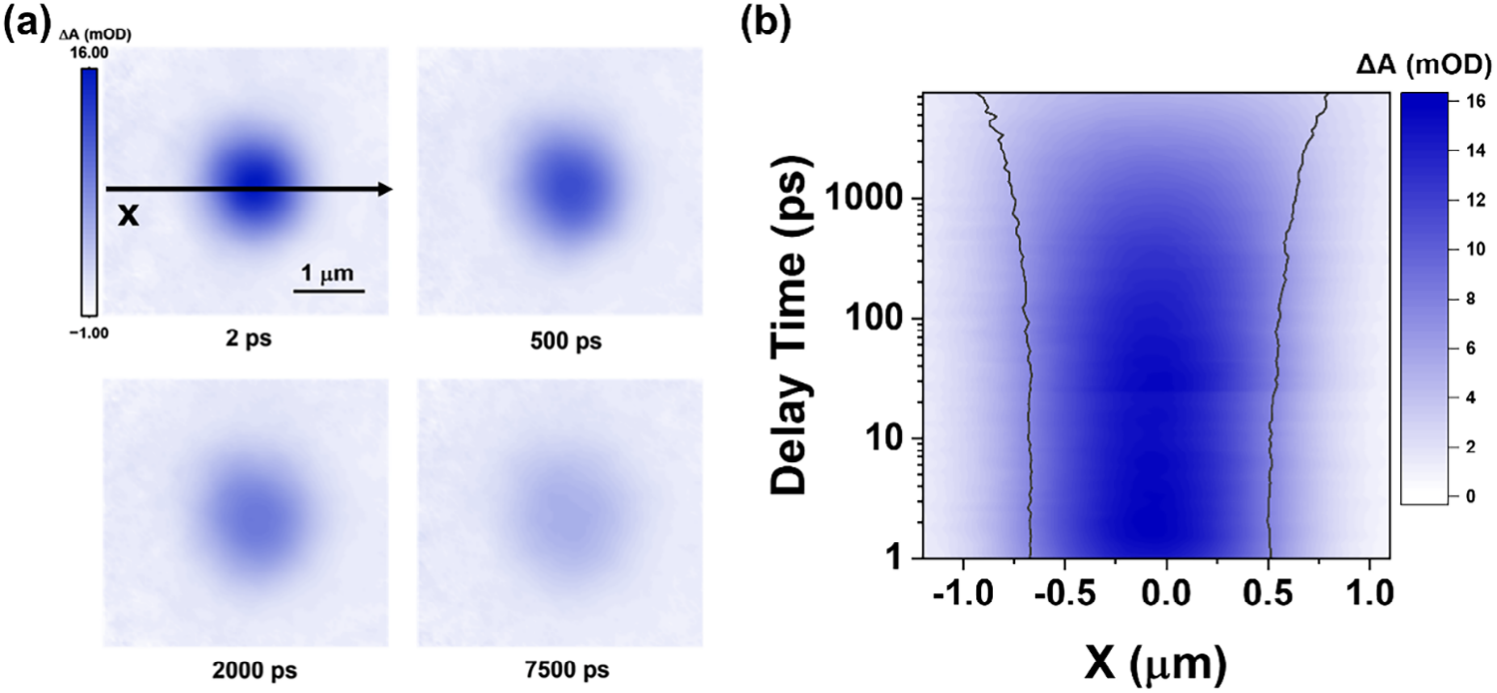
a) Diagram of the 2D imaging of the carrier density profile in PVK with an excitation power density of 27 µJ cm^−2^; b) Temporal evolution of signal intensity along the X‐axis, the solid lines represent the FWHM of the signal at different delay time.

To visualize the impact of passivation on the spatial carrier mobility, we employed TAM with an excitation pump (515 nm) at an intensity of ≈27 µJ cm^−^^2^.^[^ ^19^ ^]^ The PIA2 band, which reflects the carrier signal at the CB edge, was measured using a probe wavelength centered at 650 nm, with a specific bandwidth of 40 nm (FWHM). For morphological TAM imaging, the actinic pump focuses on a small point (≈1 µm FWHM) with the spatial intensity distribution following a Gaussian profile. The 650 nm probe beam is defocused in an area of ≈10 µm to maintain uniform light intensity in space. As the actinic pump and probe beam transmitted through the sample, carrier dynamics at the surface and bulk were collected simultaneously.^[^ ^20^ ^]^ **Figure** **5**a shows the temporal evolution of signal intensity in PVK. The TAM image at the initial time depicts the initial carrier population generated by the pump pulse. As time elapses, the TAM image showcases the diffusion of carriers away from the initial excitation volume. Figure S9 (Supporting Information) presents the kinetic traces of TAM spectra in PVK film probed at the central and edge regions of the carrier density profile. The kinetic trace in the central region exhibits an obvious decay, while distinct slow‐rising dynamics are observed when probing 500 nm away from the central region, providing evidence for significant spatial carrier expansion dynamics during the later delay time of TAM measurement.

**Figure 6:**
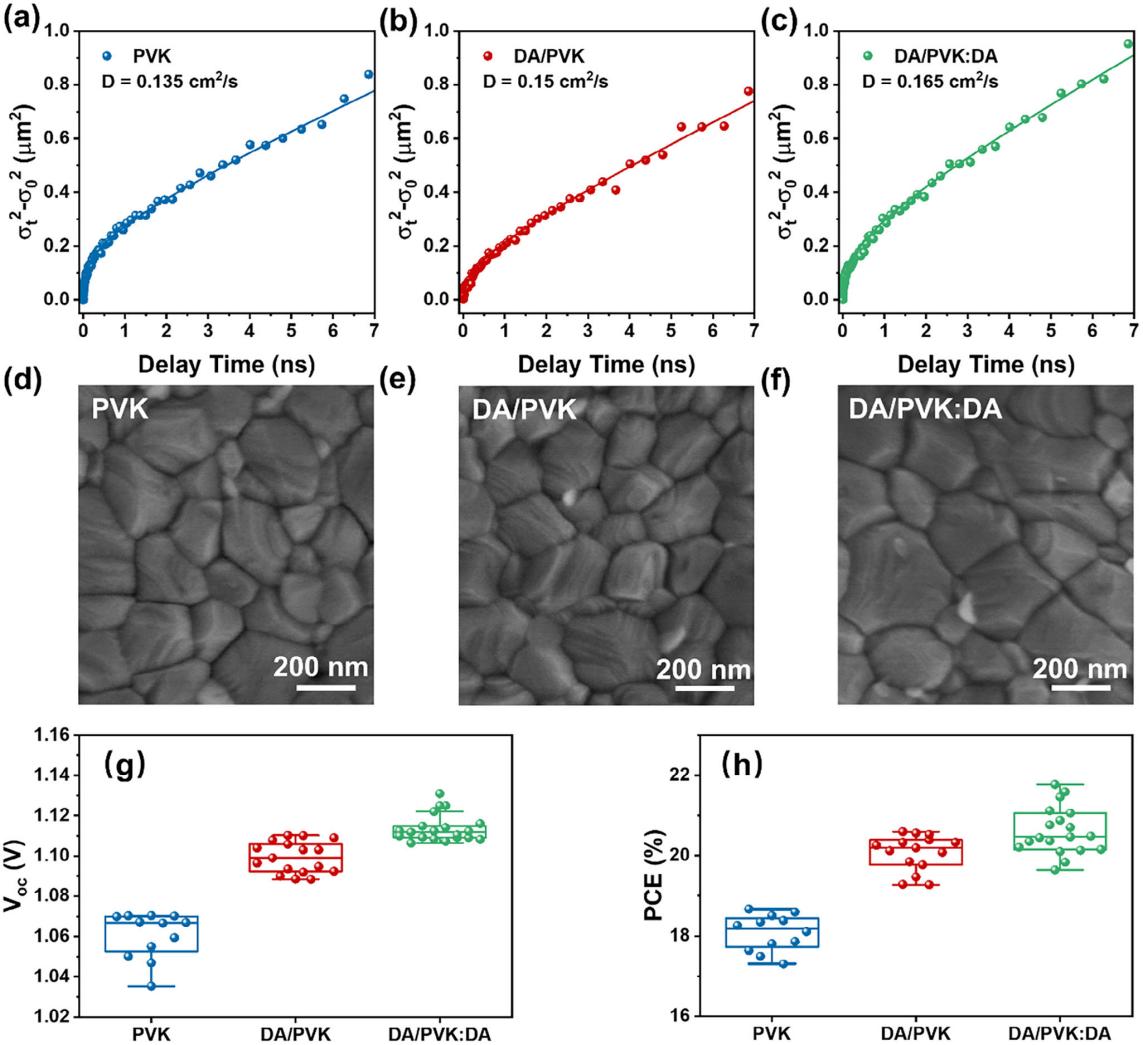
Transient σ_t_^2^‐σ_0_^2^ dynamics of a) PVK, b) DA/PVK, and c) DA/PVK:DA with an excitation power density of 27 µJ cm^−2^; The top view SEM pictures of d) PVK, e) DA/PVK and f) DA/PVK:DA, scale bar is 200 nm; Statistics of g) V_oc_ and h) PCE.

Figure 5b and Figure S10 (Supporting Information) depict the temporal evolution of the X‐axis signal intensity and the normalized spatial profiles, respectively. These profiles are well‐fitted by Gaussian functions, with the transient Gaussian parameter. σ^*2*^(representing FWHM) exhibiting a combination of nonlinear and linear broadening, as illustrated in **Figure** **6**a–c. The nonlinear broadening process can be attributed to Auger recombination and bimolecular recombination processes.^[^ ^19c^ ^]^ Transient $\sigma_{t}^{2}-\sigma_{0}^{2}$ for the three samples is well‐fitted by Equation (S1) in Support Information Section S5 (Supporting Information), depicted as the solid lines in Figure 6a–c. The diffusion constant (*D*), which represents the mobility of the carrier, is determined to be 0.135 cm^2^ s^−1^ for PVK, 0.15 cm^2^ s^−1^ for DA/PVK, and 0.165 cm^2^ s^−1^ for DA/PVK:DA, with an increase in *D* observed with further passivation. To isolate the intrinsic perovskite diffusion from transport layer effects, TAM measurements of PVK with/without a PTAA hole transport layer reveal identical diffusion constants (D = 0.13 cm^2^ s⁻¹, Figure S11, Supporting Information), confirming that TAM selectively probes in‐plane carrier dynamics unaffected by vertical extraction.

For DA/PVK, both TA and transient mid‐IR results indicate that interlayer passivation significantly reduces the DOS of the mid‐IR emissive trapping state and the SRH‐related deep defect state. As shown in Figure 6e, top‐view SEM was utilized to directly morphologies in perovskite films; it is evident that the average grain size in DA/PVK is similar to that of the pristine PVK film (Figure 6d). This suggests that DA interlayer passivation does not significantly affect the grain boundary areas or density of the trapping state in the bulk but reduces the density of the trapping state on the surface. The observed enhancement in the diffusion constant *D* can be attributed to the increased mobility of carriers on the surface, facilitated by DA interlayer passivation, which results in a smoother surface on the PVK.^[^ ^21^ ^]^ In the case of DA/PVK:DA, the top‐view SEM image reveals enlarged perovskite crystal grain sizes, as shown in Figure 6f. The additive passivation increases the activation energy and slows the crystal growth rate, resulting in larger grains.^[^ ^1^, ^22^ ^]^ Consequently, the increase in grain size reduces the grain boundary area and the density of the bulk trapping state,^[^ ^20^, ^23^ ^]^ thereby improving spatial carrier transport within the perovskite material.

Finally, to establish the correlation between carrier dynamics and device performance, we evaluate the open‐circuit voltage (V_oc_), and PCE of perovskite films with different passivation treatments, as plotted in Figure 6g,h. DA/PVK exhibits a higher V_oc_ of 1.11 V (PCE of 20.6%) compared to pristine PVK (V_oc_ of 1.07 V and PCE of 18.67%). The best performance DA/PVK: DA, which demonstrates a V_oc_ of 1.13 V and a PCE of 21.77%, is consistent with its lowest defect density and highest diffusion constant. These findings demonstrate that DA passivation significantly enhances the performance of perovskite solar cells by reducing defect‐mediated recombination and improving carrier transport.

## 3 Conclusion

The study identifies a novel mid‐IR emissive trapping state in MAPbI_3_ perovskite films, distinct from the SRH‐related defect state. Incorporating DA as an interlayer and additive effectively reduces both mid‐IR emissive trapping and SRH‐related defect states. TA, TRPL, and transient mid‐IR measurements reveal that the mid‐IR emissive defect state is linked to carrier trapping at surface defect sites, which the DA interlayer successfully addresses. The DA additive is particularly effective in mitigating bulk defects, specifically the SRH‐related defect state. Additionally, by establishing clear structure‐property relationships between defect passivation and carrier dynamics, we demonstrate that this dual‐function DA passivation not only enhances carrier mobility but also leads to substantial improvements in device performance. The key parameters are summarized in **Table** **1**. These findings highlight the critical role of minimizing surface and bulk defects to enhance the optoelectronic properties of perovskites.

**Table 1:** Key parameters of defects (mid‐IR trap and SRH defect) dynamics and device performance in DA passivated MAPbI_3_ films.

	Pristine PVK	DA/PVK	DA/PVK:DA
Mid‐IR Trap Rise (τ_2_)	0.5 ns	0.9 ns	0.8 ns
SRH Lifetime (τ_4_)	37 ns	52 ns	64 ns
Diffusion Constant (D)	0.135 cm^2^ s^−1^	0.15 cm^2^ s^−1^	0.165 cm^2^ s^−1^
V_oc_	1.07 V	1.11 V	1.13 V
PCE	18.67%	20.60%	21.77%

## Conflict of Interest

There are no conflicts of interest to declare.

## Supporting information



Supporting Information

## Data Availability

The data that support the findings of this study are available in the supplementary material of this article.
