# Supplementary material for: Surface and Bulk Defect Passivation in MAPbI3 Perovskites with Daminozide: Effects on Carrier Dynamics and Mobility
Source: Adv Sci (Weinh). 2025 May 8;12(23):2500530. doi: 10.1002/advs.202500530 (PMC12199444; doi:10.1002/advs.202500530)
Supplement: Supplementary file 1 — Supporting Information [file ADVS-12-2500530-s001.docx]

**Surface and Bulk Defect Passivation in MAPbI₃ Perovskites with Daminozide: Effects on Carrier Dynamics and Mobility**

Junhan Xie^1^, Di Li^2^, Haozheng Li^1^, Bo Peng^1^, Qinye Bao^2^, Jiaming Jiang^1†^, Bo Li^2†^, Weimin Liu^1†^

1. School of Physical Science and Technology, ShanghaiTech University, Shanghai 201210, China.
2. Department of Physics, East China Normal University, Shanghai, 200062 P. R. China

†Authors to whom correspondence should be addressed. E-mails: [liuwm@shanghaitech.edu.cn](mailto:liuwm@shanghaitech.edu.cn); [bli@ee.ecnu.edu.cn](mailto:bli@ee.ecnu.edu.cn), Jiangjm@shanghaitech.edu.cn

**1.** **Synthesis of perovskite film sample**

The perovskite precursor solution was composed of 497.8 mg lead iodide (PbI_2_, Polymer Light Technology, 99.99%) and 159.0 mg methylammonium iodide (MAI, Advanced Election Technology Co. Ltd., 99.5%) in the mixed solvent with 750 μL N, N-dimethylformamide (DMF, Sigma-Aldrich, 99.8%) and 85 μL dimethyl sulfoxide (DMSO, Sigma-Aldrich, 99.8%), To synthesize the perovskite: daminozide (DA, 99.8%) (PVK: DA) solution, 66 μL DMF was substituted by 0.05 wt% DA. The glass/ITO substrates were cleaned with detergent, deionized water, acetone, ethanol, and isopropanol by ultrasonication for 20 min, and then treated with UV-ozone for 20 min. The perovskite precursor or perovskite: daminozide solution was spin-coated at 4000 rpm for 30 s, and 150 μL chlorobenzene (CB, Sigma-Aldrich, 99.8%) as an antisolvent was pipetted onto the film after 7 s processing, followed by annealing at 100 ºC for 10 min. For the ITO/DA/PVK: DA film, a DA (2 mg/mL in methanol) layer was deposited on the substrate at 5000 rpm for 30 s, followed by annealing at 85 ºC for 5 min.^[1]^

**2. Transient Absorption Spectroscopy**

Transient absorption (TA) spectra were acquired using a commercial transient absorption spectrometer (HELIOS, Ultrafast System). The 800 nm fundamental beam was generated from a Ti:Sapphire laser system (Coherent, Astrella, 35 fs, 7 mJ/pulse, 1 kHz repetition rate). A 515 nm pulse, produced via an optical parametric amplifier (OPerA Solo, Coherent Inc.), was employed as the actinic pump for the TA measurements. The actinic pump operated at 500 Hz using an optical chopper. A broadband supercontinuum white light probe pulse, spanning a wavelength range of ~480 to ~750 nm, was generated by focusing the fundamental beam into a 2 mm CaF₂ window, with a time window limit of 7 ns.

Microsecond transient absorption (μs-TA) spectroscopy was conducted using a commercial transient absorption system (EOS, Ultrafast System). For these measurements, a 515 nm actinic pump pulse was generated from an 800 nm light source passing through an optical parametric amplifier (OPerA Solo, Coherent Inc.). The probe consisted of a broadband supercontinuum white light pulse, spanning ~350 nm to ~800 nm, which was generated by a sub-nanosecond white light laser operating at 2000 Hz.

**3. Transient Mid-infrared Spectroscopy**

The 400 nm laser pulses were generated by the second harmonic in 1 mm BBO crystal act as an actinic pump for the home-made transient mid-IR spectroscopy. The actinic pump was operated at 500 Hz by an optical chopper. Tunable mid-infrared probe pulses (4 $\mu$m to 7 $\mu$m) were generated by difference frequency mixing of signal and idler pulses from an optical parametric amplifier (OPerA Solo, Coherent Inc.). The probe pump, after collecting a sample in liquid nitrogen, cooled the mercury cadmium telluride detector, and data were collected every 500 nm. The entire pump–probe setup was purged with nitrogen gas to prevent spectral and temporal reshaping of the mid-IR pulse due to the absorption of water vapor in the air.

**4. Time-Correlated Single Photon Counting Spectroscopy**

A 1 MHz picosecond pulsed laser at 515 nm with an excitation power density of 4 $\mu$J cm^-2^ was used as the excitation pulse. Time-resolved photoluminescence (TRPL) spectroscopy was performed using the time-correlated single photon counting (TCSPC) technique, where avalanche photodiode (APD) acted as a single-photon-sensitive detector. All measurements were carried out under ambient conditions

**5. Transient Absorption Microscopy**

- **Experimental setup**


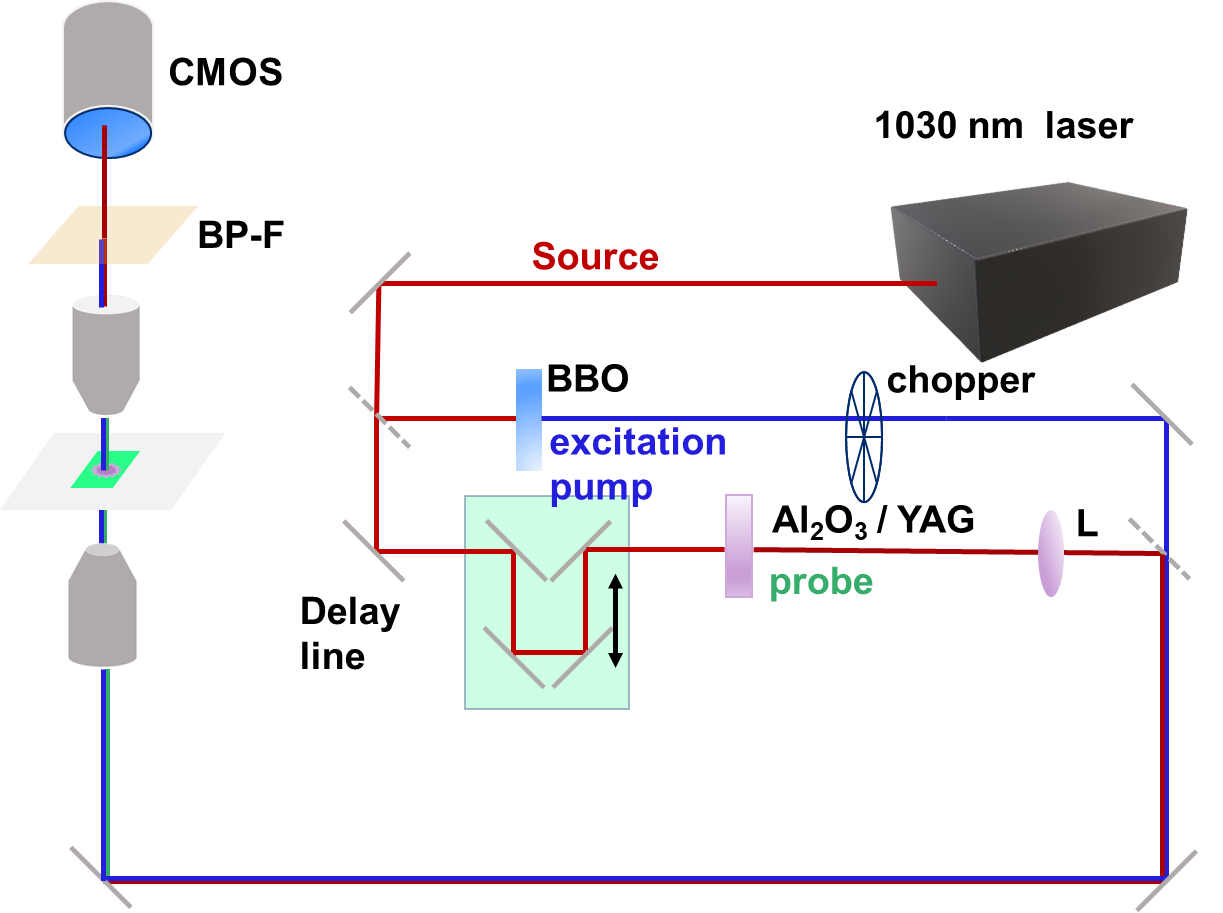


**Figure S1:** Schematic of transient absorption microscopy, L: lens, BP-F: bandpass filter.

Transient absorption microscopy (TAM) was performed using a commercial transient absorption microscope (Time-Tech Spectra, Dalian, China). The excitation source was a 1030 nm fiber laser (YactoFiber, Yili Technique, Hangzhou, China) with a pulse duration of < 290 fs and a repetition rate of 100 kHz. Second harmonic generation of the 1030 nm beam using a BBO crystal produced a 515 nm excitation pump. A chopper operating at 1000 Hz was placed in the pump beam path to modulate the beam. The probe pulse was generated as a broadband white light continuum (~400 nm to ~900 nm) by focusing the 1030 nm beam onto a 2 mm sapphire plate.

The pump and probe beams were combined using a long-pass filter and directed toward a microscope. The pump beam was focused onto the sample through a microscope objective (Olympus, 100× magnification, numerical aperture of 0.5, working distance of 1 mm), producing a focused spot with FWHM of ~1 μm on the sample surface. The probe beam was expanded using a f = 300 mm focal length lens, creating a wide-field illumination with a ~10 μm FWHM spot size. The transmitted probe light was collimated and focused onto a charged metal-oxide-semiconductor (CMOS) detector (Basler acA640) using another 100× microscope objective. A band-pass filter (centered at 650 nm, FWHM of 40 nm) was employed to isolate the desired signal wavelength. The CMOS detector, with a resolution of 360 × 480 pixels, captured the transmitted light. Each pixel corresponded to an area of 50 nm × 50 nm under the 100× magnification of the microscope.

- **TAM experimental data processing:**

The evolution of carrier population in 2-D space and time can be described by Equation (1)^[2]^

$$\begin{aligned} \frac{\partial n\left( x,y,t \right)}{\partial t}=D\left( \frac{\partial^{2}n\left( x,y,t \right)}{\partial x^{2}}+\frac{\partial^{2}n\left( x,y,t \right)}{\partial y^{2}} \right)-\frac{n\left( x,y,t \right)}{\tau}-\gamma n^{2}\left( x,y,t \right)-\eta n^{3}\left( x,y,t \right)\#\left( 1 \right) \end{aligned}$$

In this context, n(x, y, t) represents the carrier population as a function of position (x, y) and delay time (t). Assuming a linear proportionality between the transient absorption (TA) signal and the carrier population density, the transient absorption microscopy (TAM) experiments allow for the temporal mapping of the spatial distribution of carrier populations. The carrier lifetime τ, which reflects the combined responses of electrons and holes, is expected to contribute equally to the diffusion process due to the similar effective masses and SRH lifetimes of carriers. The diffusion constant D represents the mobility of the carrier. Additionally, γ and η represent the high-order coefficients for bimolecular recombination and Auger recombination, respectively. In this work, γ and η are constant, which are not affected by passivation, hence γ = 5×10^-12^ cm^3^/s and η = 5.5 ×10^-27^ cm^6^/s are used to fit.^[3]^ At t = 0, the excitation pump beam exhibits a Gaussian spatial distribution, leading to an initial carrier population density, n(x, y, 0), that follows a Gaussian distribution. Hence, the initial carrier distribution along the X-axis can be expressed as:

$$\begin{aligned} n\left( x,0 \right)=\frac{1}{\sigma_{0}\sqrt{\pi/2}}e^{-\frac{x^{2}}{\sigma_{0}^{2}}}\#\left( 2 \right) \end{aligned}$$

According to initial distribution condition in Equation (2), the general solution of first-order Equation (1) can be described as a Gaussian function

$$\begin{aligned} n\left( x,t \right)=\frac{1}{\sigma_{t}\sqrt{\pi/2}}e^{-\frac{x^{2}}{\sigma_{t}^{2}}}\#(3) \end{aligned}$$

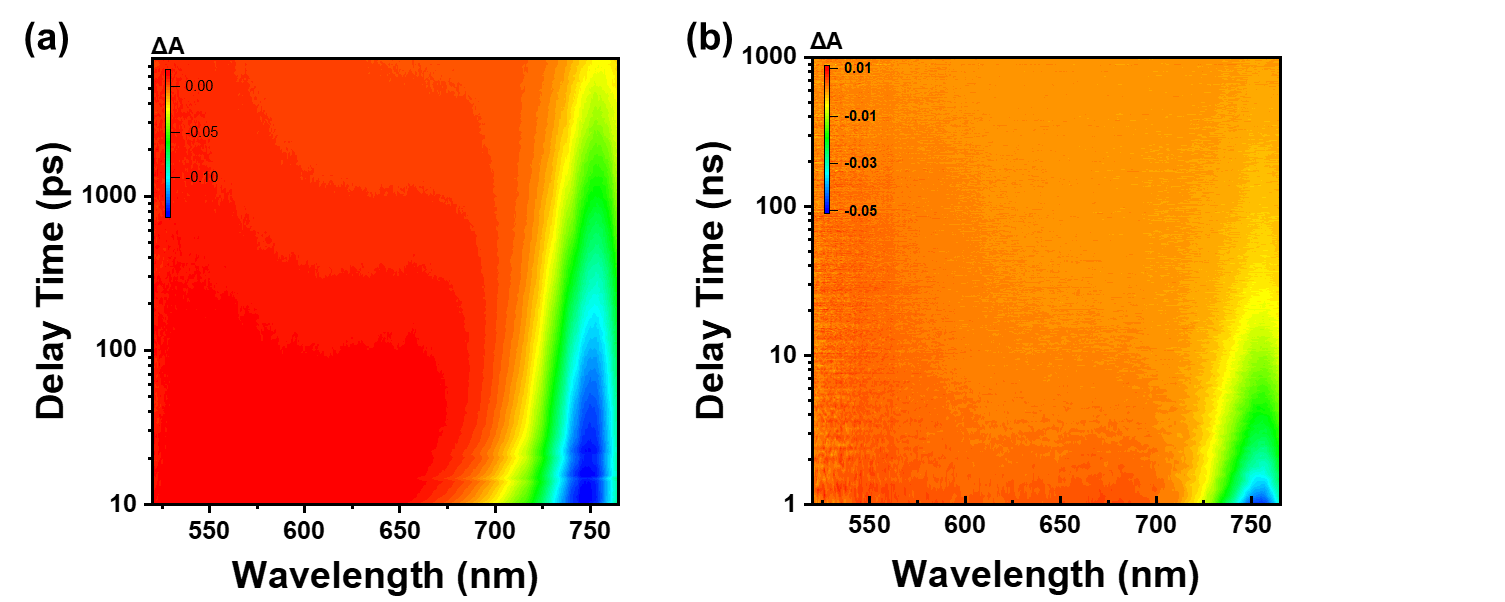


**Figure S2:** 2D plot of (a) TA spectrum and (b) μs-TA spectrum of PVK under 515 nm excitation with the actinic pump power density of 128 μJ/cm^2^.


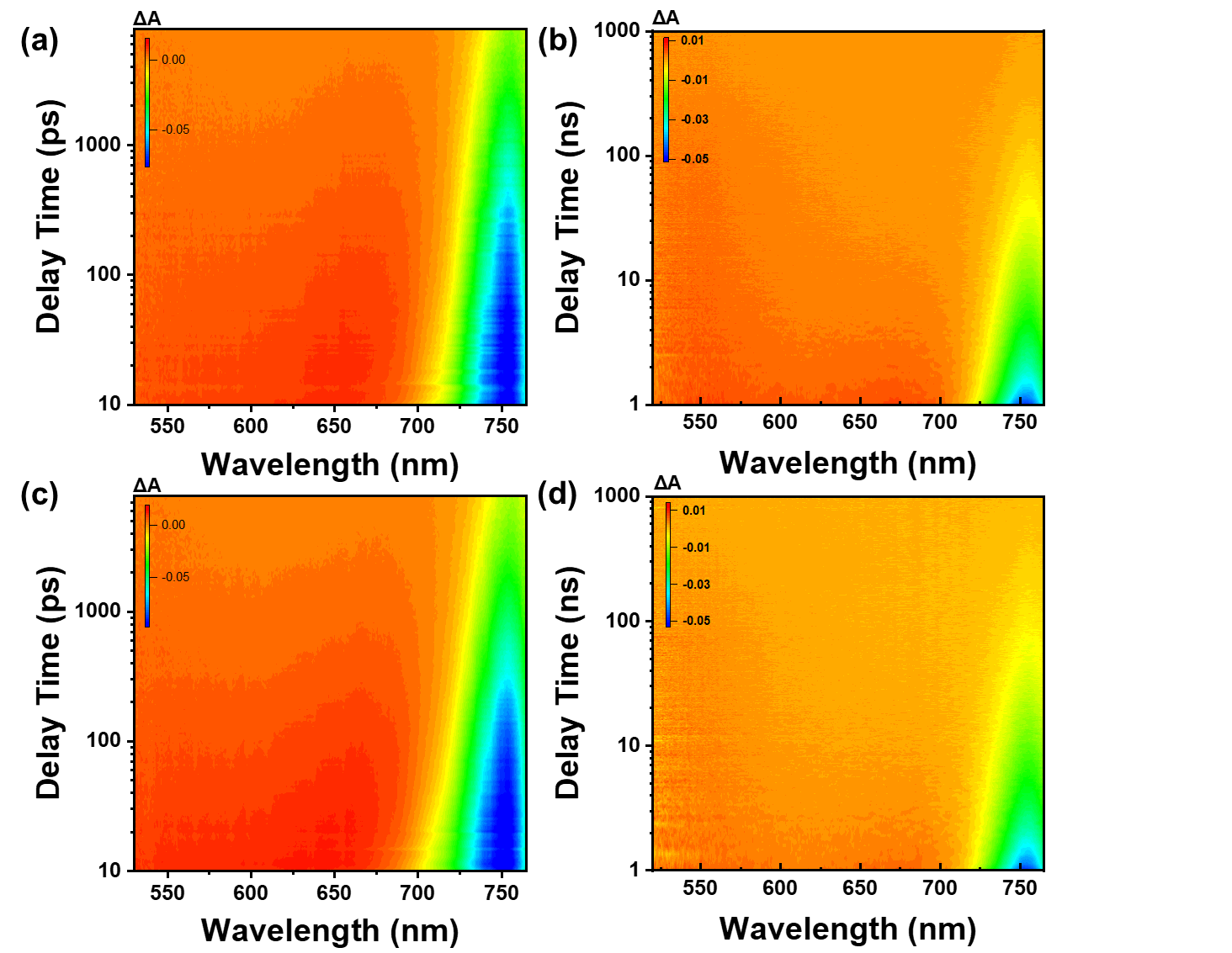


**Figure S3:** 2D plot of TA spectrum of (a) DA/PVK and (c) DA/PVK:DA under 515 nm excitation with the actinic pump power density of 128 μJ/cm^2^; 2D plot of μs-TA spectrum of (b) DA/PVK and (d)DA/PVK:DA under 515 nm excitation with the actinic pump power density of 128 μJ/cm^2^.

**
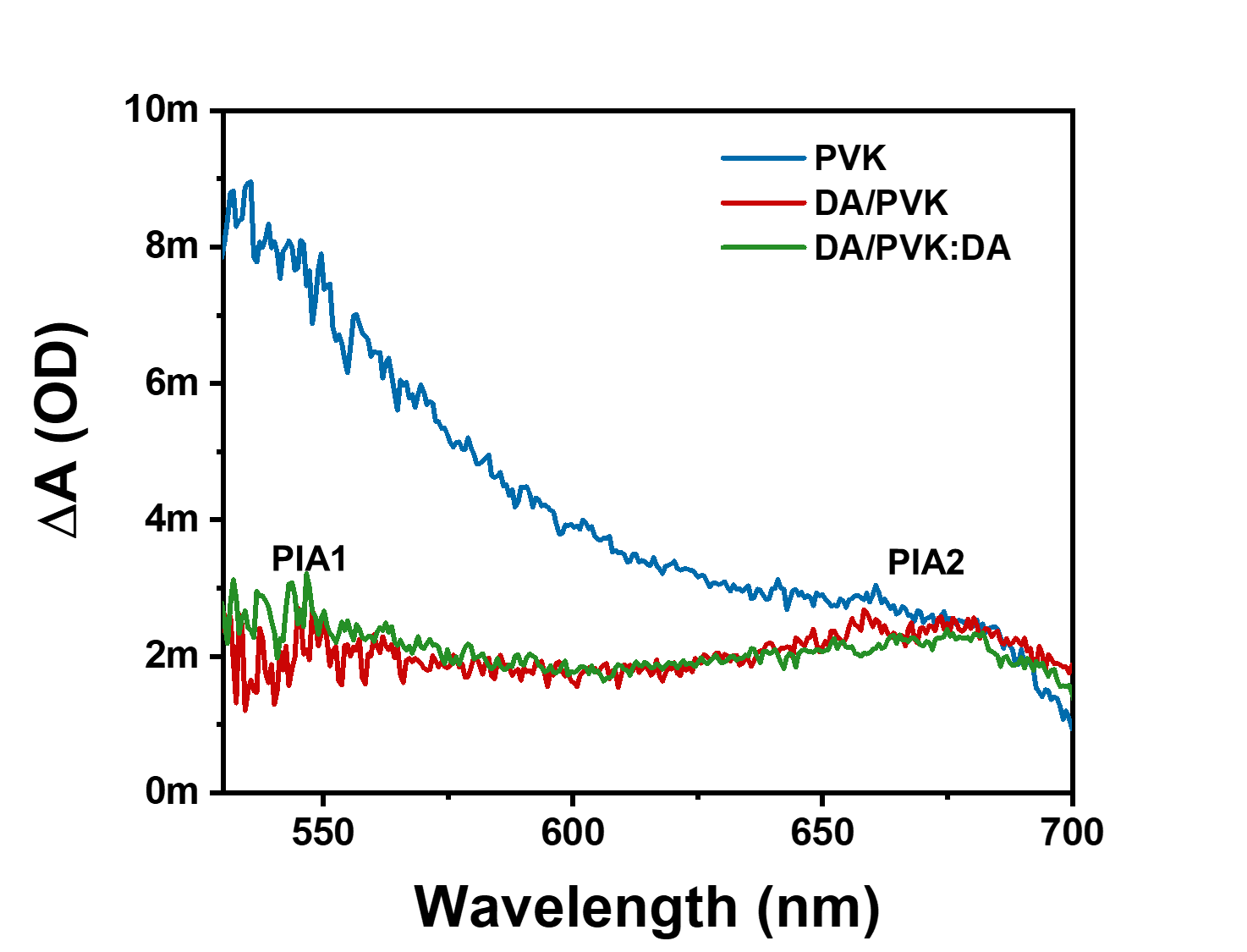
**

**Figure S4:** TA spectra of PVK, DA/PVK, and DA/PVK:DA at the delay time of 4 ns.


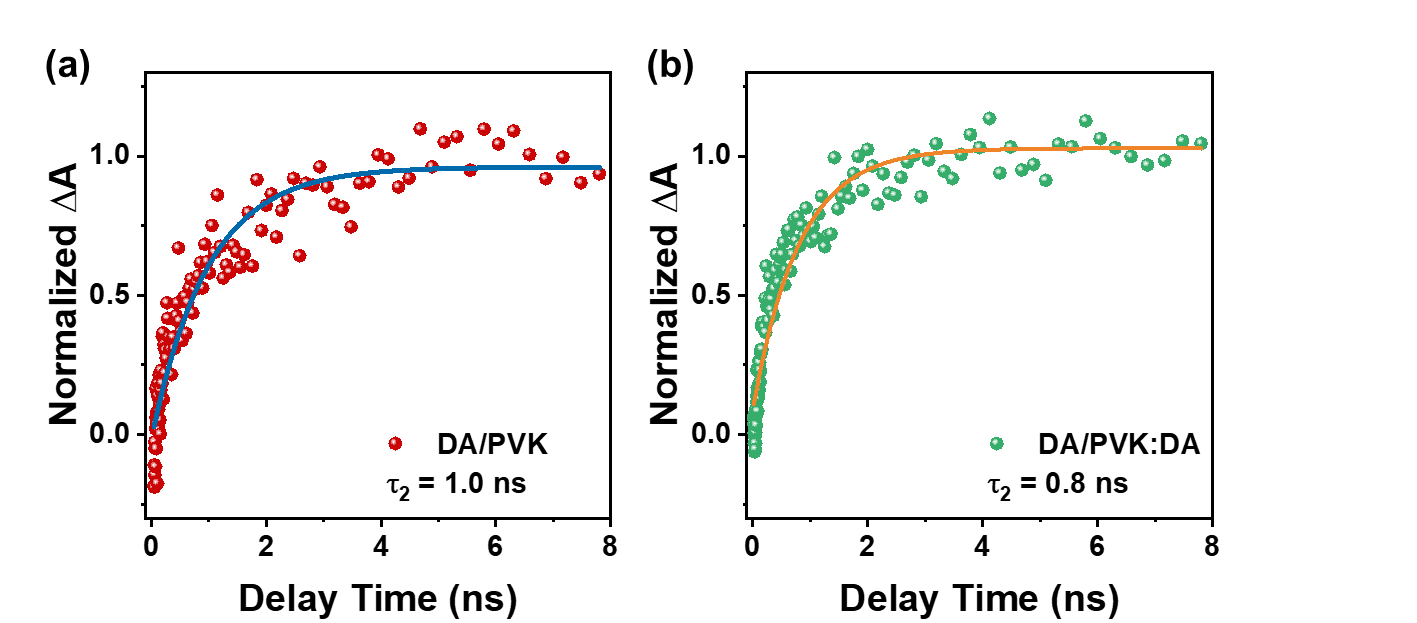


**Figure S5:** The normalized rising dynamics of the sub-bandgap trapping state (mid-IR emissive trapping state) within PIA1 band of (a)DA/PVK and (b)DA/PVK:DA.


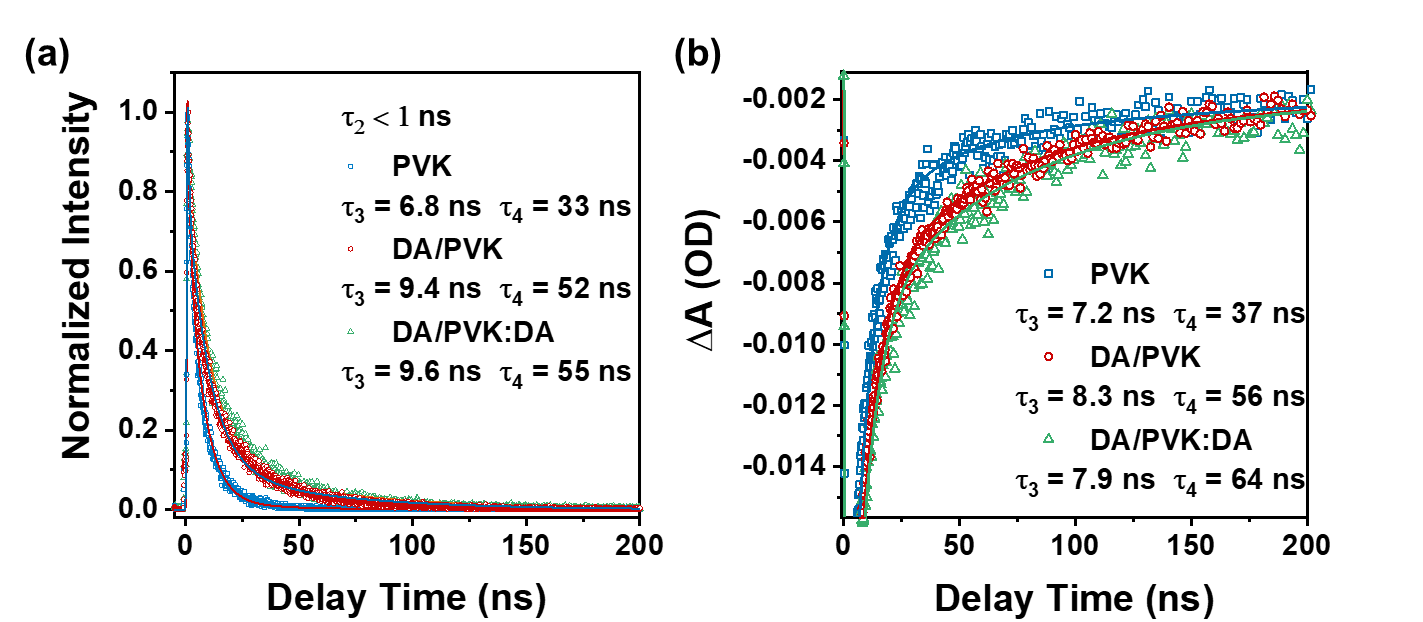


**Figure S6:** (a)Kinetic profiles of TRPL in PVK, DA/PVK, and DA/PVK:DA under 4 μJ/cm^2^ excitation, IRF = 0.5 ns; (b) Kinetic profiles of GSB signal from TA in PVK, DA/PVK, and DA/PVK:DA under 128 μJ/cm^2^ excitation.


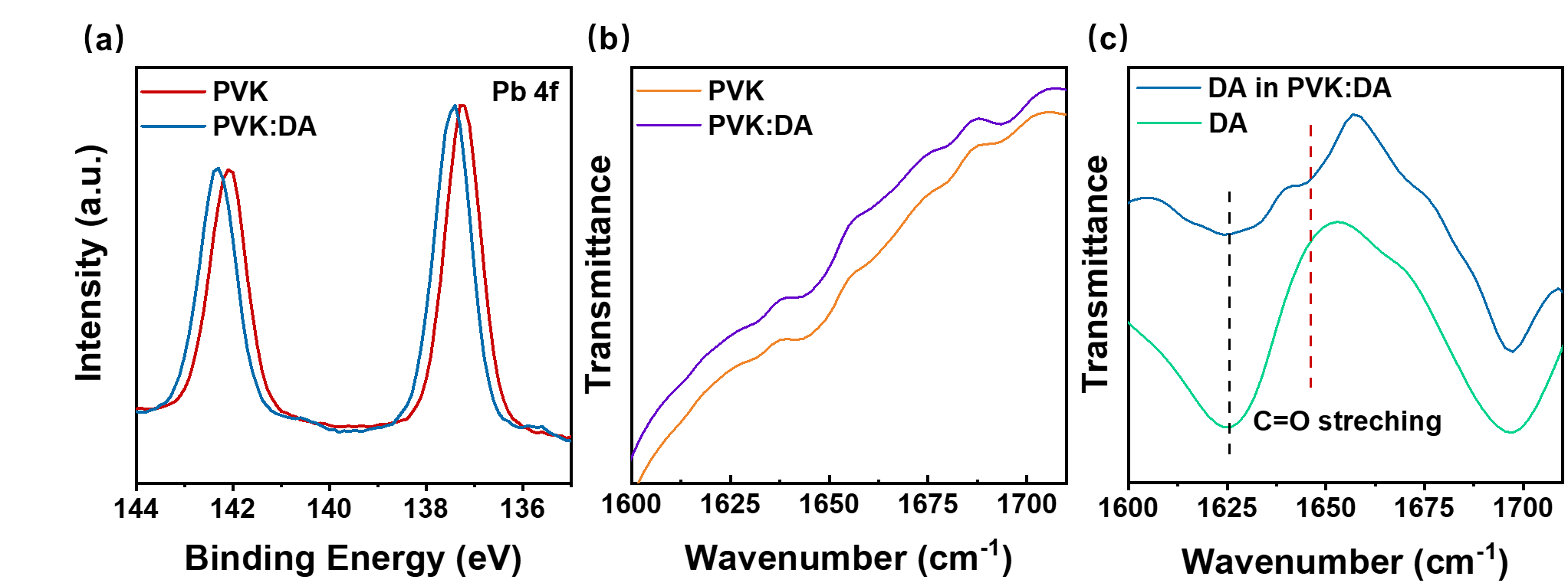


**Figure S7:** (a) X-ray photoelectron spectroscopy (XPS) of PVK and PVK:DA. (b) Fourier transform infrared spectroscopy (FTIR) of PVK and PVK:DA (c) FTIR spectra of DA extracted from PVK:DA (obtained by subtracting PVK spectra from PVK:DA in (b) ) and pristine DA, highlighting the C=O stretching region.


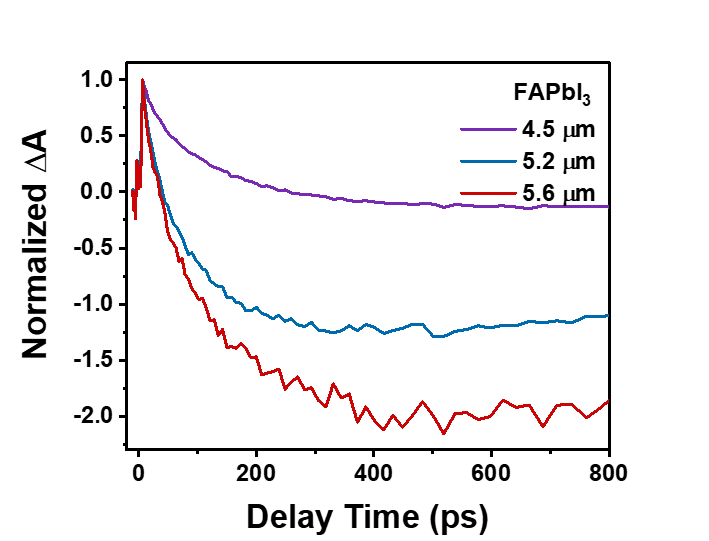


**Figure S8:** Normalized transient mid-IR trace of FAPbI_3_ at 4.5 μm, 5.2 μm, and 5.6 μm.

**
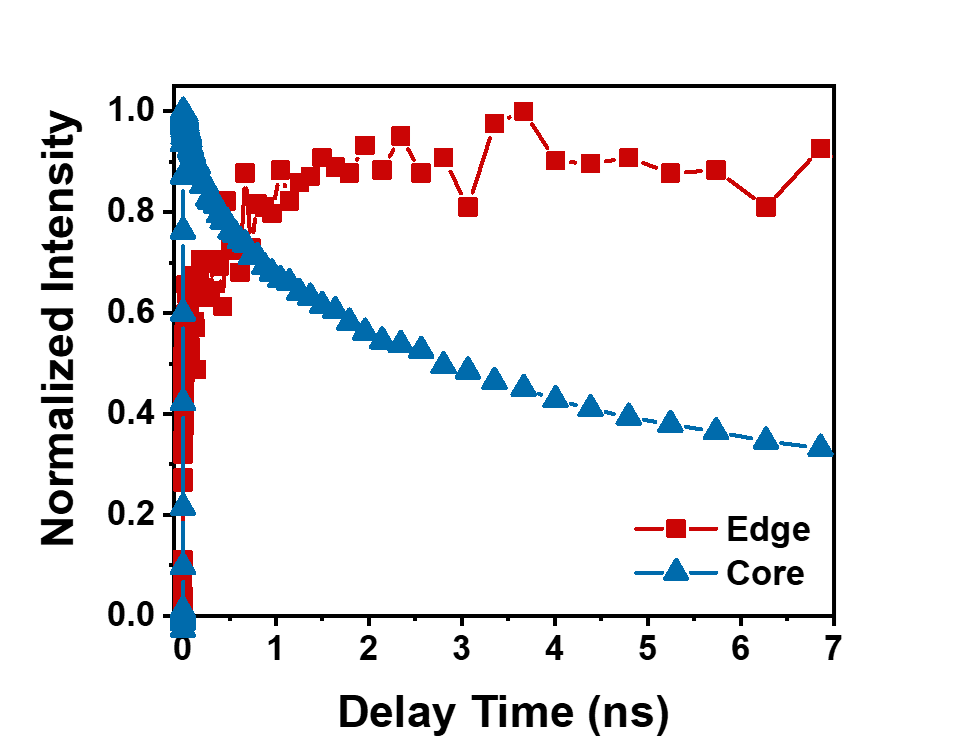
**

**Figure S9:** (a) Kinetic traces of TAM spectra in PVK film probed at the central (blue triangle) and edge regions (500 nm away from the center, red square) of the carrier density profile.


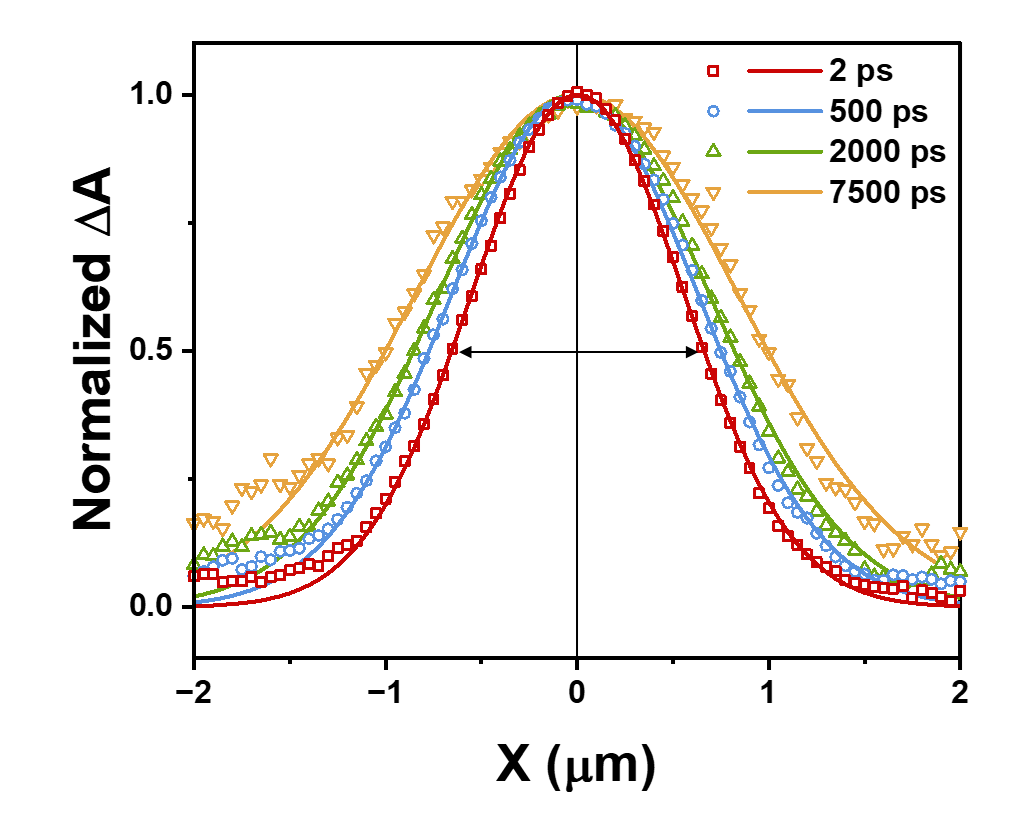


**Figure S10:** Normalized X-axis TAM signal at different delay times. Solid lines represent Gaussian function fits.


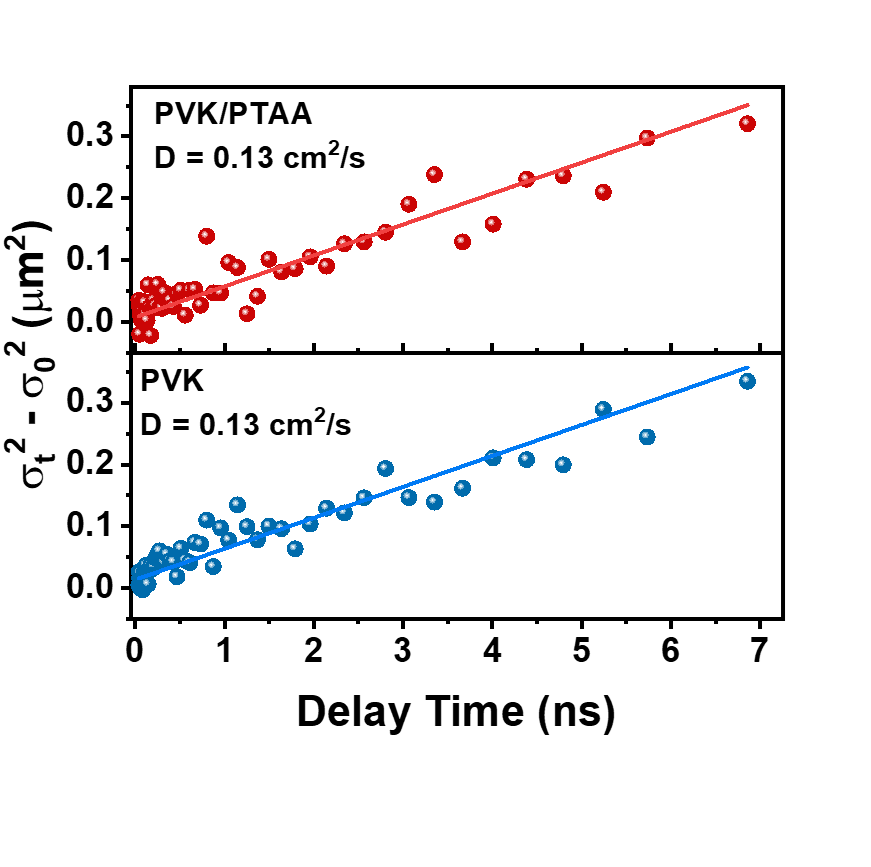


**Figure S11:** Transient σ_t_^2^-σ_0_^2^ dynamics of PVK/PTAA and PVK under 7 μJ cm^-2^ excitation.

**Reference**

.[1] Y. H. Xu, S. B. Xiong, S. Jiang, J. M. Yang, D. Li, H. B. Wu, X. M. You, Y. F. Zhang, Z. F. Ma, J. H. Xu, J. X. Tang, Y. F. Yao, Z. R. Sun, Q. Y. Bao, *Adv. Energy Mater.* **2023**, 13, 2203505.

[2] Z. Guo, J. S. Manser, Y. Wan, P. V. Kamat, L. B. Huang, *Nat. Commun.* **2015**, 6, 7471.

[3] J. H. Xie, W. Zhou, H. Z. Li, Z. Y. Wang, J. M. Jiang, Y. L. Zhang, X. Q. Shen, Z. J. Ning, W. M. Liu, *Adv. Opt. Mater.* **2024**, 12, 2303004.
